# Severe Rhabdomyolysis Due to Strenuous Exercise With a Potential Role of a High-Caffeine Energy Drink

**DOI:** 10.7759/cureus.20867

**Published:** 2022-01-01

**Authors:** Mohammad Tinawi

**Affiliations:** 1 Nephrology, Nephrology Specialists, Munster, USA; 2 Medicine, Indiana University School of Medicine Northwest-Gary, Gary, USA

**Keywords:** exercise, caffeine, intravenous fluids, exercise-induced rhabdomyolysis, energy drinks

## Abstract

Caffeine is the core ingredient in energy drinks. These drinks are commonly consumed by athletes around the time of their workout to boost energy levels. The patient in the case presented is a 40-year-old-man who developed severe rhabdomyolysis after consuming an energy drink with high content of caffeine prior to a strenuous workout. He was successfully treated with isotonic intravenous solutions. Clinicians should be aware of the potential adverse reactions associated with the use of energy drinks.

## Introduction

Rhabdomyolysis requires immediate medical attention because it is potentially life-threatening. Necrosis of striated muscles leads to the release of potentially toxic intracellular constituents such as creatine kinase (CK), myoglobin, potassium, and phosphate [1]. Common causes include muscle trauma including crush injuries, strenuous exercise, medications (especially statins), hypophosphatemia, hypokalemia, infections, malignant hyperthermia, and certain genetic disorders [2]. CK-MM subtype is the most sensitive diagnostic test. CK rises within a few hours of the muscle injury and peaks after 24-72 hours.

Many athletes consume energy drinks to boost their performance. Adverse reactions due to energy drinks have been reported. The energy drink market is expanding quickly. Brandessence® Market Research reported that the size of the energy drink market was USD 61.23 billion in 2020 and is expected to reach USD 99.62 by 2027 [3]. The main ingredient in energy drinks is caffeine [4].

## Case presentation

The patient is a 40-year-old-man who exercises regularly. His routine workouts are strenuous and involve heavy weightlifting. In the workout preceding his hospital admission, he was lifting more than what is customary for him. He also drank 355 mL (12 oz) of a popular energy drink. A few hours after his exercise, he noted that his urine became cola-colored. He started to hydrate orally with tap water. He developed diffuse arthralgia and myalgia particularly neck pain. He presented to the emergency department (ED) four days after his exercise session. His past medical and surgical history was unremarkable. He is a non-smoker, non-drinker, and does not use illicit drugs. His family history is negative for chronic kidney disease.

His vital signs were stable and unremarkable. His physical examination showed a healthy-appearing man with a large muscle mass, weight of 100.8 kg, height of 173 cm, and BMI of 33.8. The examination was unremarkable. His medications included cyclobenzaprine 10 mg twice daily and tramadol 50 mg three times daily. He was prescribed these two medications a year ago for neck pain and took them for only a few days then. He restarted taking cyclobenzaprine and tramadol when his myalgia started four days prior to his ED presentation.

His creatinine has been stable in the range of 1.2-1.4 mg/dL since 2016. Electrolytes, blood sugar, and hemoglobin A1C have been in the normal range over the same period. His elevated creatinine appeared consistent with his large muscle mass in the absence of any history of hypertension, diabetes mellitus, proteinuria, hematuria, cystic kidney disease, or family history of chronic kidney disease. In the ED, his labs were as follows: sodium of 140, potassium of 4.2, chloride of 104, serum CO_2_ of 28 (all in mEq/L); blood urea nitrogen of 28, creatinine of 1.38, glucose of 99, and calcium of 9.4 (all in mg/dL); and hemoglobin of 15.9 g/dL. Initial CK was 139,680 U/L (reference range: 20-200 U/L), serum myoglobin was 13,538 ng/mL (reference range: 28-72 ng/mL), and urine myoglobin was 471 ng/mL (reference range: 0-13 ng/mL). Urinalysis showed brown urine, specific gravity of 1.013, pH of 5.5, protein of 100 mg/dL, blood 3+, RBCs of 3 per high power field (HPF), WBCs of 5/HPF, 1 epithelial cast/HPF, and no glucose, ketone, bilirubin, or nitrite. Urine drug screen was completely negative.

The patient was admitted to the hospital with a diagnosis of severe rhabdomyolysis. He was given 2 L of isotonic saline in the ED followed by an isotonic sodium bicarbonate intravenous (IV) solution (150 mEq sodium bicarbonate in 1 L of 5% dextrose at 150 mL/hour or 3.6 L daily). While on this IV solution, his urine pH rose to 9.0. He stayed in the hospital for eight days on the above IV solution, and his CK was checked daily. Daily laboratory values are summarized in Table 1.

**Table 1:** CK and other significant laboratory tests per hospital day. CK, creatine kinase; Na, sodium; K, potassium; Ca, calcium; CO_2_, serum CO_2_; Cr, creatinine

Day	1	2	3	4	5	6	7	8
CK (U/L)	139,680	139,250	101,430	75,696	43,700	15,762	9,413	5,634
Na (mEq/L)	140	140	142	141	142	140	142	143
K (mEq/L)	4.2	3.4	3.3	3.3	3.5	3.5	3.5	3.4
Ca (mg/dL)	9.4	9.0	9.0	9.5	9.5	9.5	9.4	9.5
CO_2_ (mEq/L)	28	34	34	34	34	36	34	32
Cr (mg/dL)	1.38	1.5	1.47	1.47	1.48	1.55	1.49	1.46

The patient’s creatinine remained remarkably stable in the range of 1.38-1.55 mg/dL. Serum CO_2_ was in the range of 28-36 mEq/L and was 32 mEq/L at the time of discharge. He received potassium chloride orally for a serum potassium of 3.3 mEq/L on hospital days 3 and 4. His hypokalemia was due to the administration of potassium-free IV fluids, and intracellular potassium shift was due to sodium bicarbonate. The sodium bicarbonate isotonic solution resulted in serum CO_2_ elevation. Serum calcium remained normal. The patient did not require pain medications. At the time of his discharge, CK was down to 5,634 U/L, and it was felt that it would be safe to discontinue IV fluids. He was discharged home in a stable condition, with instructions to avoid strenuous physical activities for two weeks.

## Discussion

Rhabdomyolysis initial treatment involves aggressive hydration with IV fluids to prevent acute kidney injury and severe life-threatening hyperkalemia. Hyperkalemia should be promptly treated with medical measures. Hemodialysis should be initiated without delay in case of severe life-threatening hyperkalemia [3].

The patient added to 355 mL (12 fl oz) of water one serving of a popular energy drink containing caffeine anhydrous 300 mg, dicaffeine malate 100 mg, citrulline malate 6,000 mg, beta-alanine 3,200 mg, taurine 1,000 mg, agmatine sulfate 1,000 mg, and cocophenols 25 mg. These ingredients are common in energy drinks [4]. The dose of caffeine received was 4 mg/kg. A 237-mL (8 oz.) cup of brewed black coffee contains approximately 100 mg of caffeine, and the same amount of brewed black tea and caffeinated cola contain approximately 50 mg and 24 mg of caffeine, respectively [5]. Caffeine in energy drinks is usually a synthetic alkaloid rather than naturally occurring as in tea or coffee. Caffeine is rapidly absorbed and peaks 30-60 minutes post-ingestion [6]. Caffeine is used by many athletes as a stimulant and performance enhancer, although this effect remains a subject of controversy [7]. Energy drinks have been associated with blood pressure elevation, tachycardia, cardiac arrhythmias, and even sudden cardiac death. Direct causation has not been proven [8]. Energy drinks have also been associated with rhabdomyolysis [9,10]. The lethal dose of caffeine is 10 g or higher [6]. This is significantly more than the 400 mg dose ingested by this patient. Although the pathogenesis of caffeine-induced rhabdomyolysis is unclear, several mechanisms have been proposed. At therapeutic levels, caffeine stimulates β1,2 receptors with subsequent release of catecholamines. At high levels, caffeine inhibits cyclic adenosine monophosphate esterases, resulting in further dopaminergic sensitization, β-adrenergic stimulation, and rise in serum calcium [6]. Other mechanisms include an increase in intracellular calcium, which causes muscle contractions that lead to injury, and caffeine-induced muscle fasciculations and irritability [11]. The patient in this case started oral hydration prior to his hospitalization and then received hydration with isotonic IV solutions during his hospital stay with adequate urine alkalinization. The use of sodium bicarbonate solutions for hydration in rhabdomyolysis remains controversial; moreover, there is no evidence from clinical trials that they are superior to isotonic saline [12]. Therefore, the focus should be on early and aggressive volume resuscitation with isotonic saline, which is readily available. Aggressive hydration prevented acute kidney injury and the need for renal replacement therapy in this patient. Despite the severity of his rhabdomyolysis, he did not develop any significant complications. This patient does not have a prior history of rhabdomyolysis, and he has not been consuming energy drinks until this incident. In the absence of any other risk factors for rhabdomyolysis, it is conceivable that the energy drink combined with strenuous exercise have increased the risk of rhabdomyolysis. The high content of caffeine is likely to induce diuresis and dehydration. Proving causation remains difficult and would require clinical trials with significant resources. Consumption of energy drinks is not recommended in children and adolescents [13].

## Conclusions

The case presented illustrates the increased risk of rhabdomyolysis in athletes who consume energy drinks in conjunction with strenuous exercise. High caffeine content of energy drinks may enhance the risk of rhabdomyolysis; however, direct causation remains unproven. Early and aggressive resuscitation with normal saline is paramount in patients presenting with rhabdomyolysis. This approach stabilized renal function in our patient despite the severity of his rhabdomyolysis.
